# Dissection of transcriptomic and epigenetic heterogeneity of grade 4 gliomas: implications for prognosis

**DOI:** 10.1186/s40478-023-01619-5

**Published:** 2023-08-14

**Authors:** Chang Zeng, Xiao Song, Zhou Zhang, Qinyun Cai, Jiajun Cai, Craig Horbinski, Bo Hu, Shi-Yuan Cheng, Wei Zhang

**Affiliations:** 1 Department of Preventive Medicine, Northwestern University Feinberg School of Medicine, 680 N. Lake Shore Dr., Suite 1400, Chicago, IL 60611 USA; 2 The Ken and Ruth Davee Department of Neurology, Northwestern University Feinberg School of Medicine, 303 E. Chicago Ave., Chicago, IL 60611 USA; 3 Huashan Hospital, Fudan University, 12 Wulumuqi Rd., Shanghai, 200040 China; 4 Department of Pathology, Northwestern University Feinberg School of Medicine, 303 E. Chicago Ave., Chicago, IL USA; 5 The Robert H. Lurie Comprehensive Cancer Center and Simpson Querrey Institute for Epigenetics, Northwestern University Feinberg School of Medicine, 303 E. Chicago Ave., Chicago, IL 60611 USA

**Keywords:** Grade 4 glioma, Glioblastoma, Heterogeneity, Epigenetics, 5-Hydroxymethylcytosine, Prognosis

## Abstract

**Background:**

Grade 4 glioma is the most aggressive and currently incurable brain tumor with a median survival of one year in adult patients. Elucidating novel transcriptomic and epigenetic contributors to the molecular heterogeneity underlying its aggressiveness may lead to improved clinical outcomes.

**Methods:**

To identify grade 4 glioma -associated 5-hydroxymethylcytosine (5hmC) and transcriptomic features as well as their cross-talks, genome-wide 5hmC and transcriptomic profiles of tissue samples from 61 patients with grade 4 gliomas and 9 normal controls were obtained for differential and co-regulation/co-modification analyses. Prognostic models on overall survival based on transcriptomic features and the 5hmC modifications summarized over genic regions (promoters, gene bodies) and brain-derived histone marks were developed using machine learning algorithms.

**Results:**

Despite global reduction, the majority of differential 5hmC features showed higher modification levels in grade 4 gliomas as compared to normal controls. In addition, the bi-directional correlations between 5hmC modifications over promoter regions or gene bodies and gene expression were greatly disturbed in grade 4 gliomas regardless of *IDH1* mutation status. Phenotype-associated co-regulated 5hmC–5hmC modules and 5hmC–mRNA modules not only are enriched with different molecular pathways that are indicative of the pathogenesis of grade 4 gliomas, but also are of prognostic significance comparable to *IDH1* mutation status. Lastly, the best-performing 5hmC model can predict patient survival at a much higher accuracy (c-index = 74%) when compared to conventional prognostic factor *IDH1* (c-index = 57%), capturing the molecular characteristics of tumors that are independent of *IDH1* mutation status and gene expression-based molecular subtypes.

**Conclusions:**

The 5hmC-based prognostic model could offer a robust tool to predict survival in patients with grade 4 gliomas, potentially outperforming existing prognostic factors such as *IDH1* mutations. The crosstalk between 5hmC and gene expression revealed another layer of complexity underlying the molecular heterogeneity in grade 4 gliomas, offering opportunities for identifying novel therapeutic targets.

**Supplementary Information:**

The online version contains supplementary material available at 10.1186/s40478-023-01619-5.

## Background

WHO (World Health Organization) grade 4 gliomas including *IDH* wild-type glioblastoma (GBM) and *IDH* mutant astrocytoma are the most malignant primary tumors in the Central Nervous System (CNS) [1]. Grade 4 glioma is diagnosed in ~ 13,000 new patients with ~9000 associated deaths in the United States every year [2]. Despite advances in surgery and combination therapies, clinical outcomes for grade 4 gliomas have not been significantly improved [3]. A hallmark of these malignant brain tumors is their heterogeneity, wherein discrete subsets of grade 4 gliomas display unique patterns of pathogenesis, biology, and prognosis [3, 4]. Since the microenvironment within a tumor is not homogeneous, differences in oxygen pressure, blood vessel density, growth factors, and composition of extracellular matrix occur naturally in tumors, which in turn may manifest phenotypic and mutational/epigenetic differences [5]. The intrinsic and extrinsic heterogeneity together with the brain-exclusive microenvironment result in reduced therapeutic response and uniformly poor prognosis among patients with grade 4 gliomas [4, 6–9]. Particularly, increased heterogeneity in grade 4 gliomas is known to be associated with poor prognosis with worse overall survival (OS) [6–9]. Tumor heterogeneity thus has direct translational relevance in guiding therapeutic strategies. While specific molecular markers such as mutations in the genes encoding *IDH1* (isocitrate dehydrogenase 1) and *EGFR* have been implicated in clinical diagnosis, prognosis and treatments, further improvement in prognostic stratification and novel therapies are still urgently needed to improve clinical outcomes [10].

Previous studies have revealed perturbation within the cancer epigenome, the mediator between environment and genome, and a common cancer hallmark as well [11]. Epigenetic factors such as cytosine modifications, histone modifications, and various non-coding RNAs (ncRNAs), as key regulators, play critical roles in the development and progression of tumorigenesis [11–13]. In grade 4 gliomas, methylation status of *MGMT* (O-6-Methylguanine-DNA Methyltransferase) gene promoter has been identified as a robust and independent predictive factor for the response to temozolomide, the first-line chemotherapy for grade 4 gliomas [14]. Therefore, elucidating novel epigenetic modifications that reflect the heterogeneity of grade 4 gliomas and their interactions with gene transcription could enhance our understanding of the underlying mechanisms of prognosis and responses to treatments in grade 4 gliomas.

Distinct from the extensively studied 5-methylcytsoines (5mC) in cancers, 5-hydroxymethylcytosines (5hmC) are epigenetic modifications enriched primarily in enhancers as well as gene bodies and promoters of actively expressed genes [15]. Depletion of 5hmC has been associated with the hypermethylation of gene bodies in various cancers including grade 4 gliomas [16–21]. Despite the positive correlation between 5hmC level and gene expression, a recent study in colorectal cancer also revealed a positive association between 5hmC and lncRNA transcription [22]. However, the interactions between 5hmC and transcriptomic features in grade 4 gliomas remain to be explored. In addition, accumulating studies showed the association between 5hmC and heterogeneity and the clinical outcomes in grade 4 gliomas, therefore suggesting 5hmC as novel epigenetic biomarkers for improved stratification in patients with grade 4 gliomas [19, 23]. However, previous studies were restricted to functional relevance of 5hmC in the gene body regions. Extending co-localization analysis between 5hmC modification and gene body regions to other genomic features such as promoters and histone modifications will likely offer opportunities for identifying therapeutic targets and prognostic markers. Because 5hmC dynamics can be informative of gliomagenesis and is highly tissue-specific, an epigenome-wide analysis of 5hmC in grade 4 gliomas will improve our understanding of the interactions between 5hmC and transcriptional products as well as the implications of 5hmC in response to therapies and patient survival in grade 4 gliomas [24–28].

In this study, we explored the prognostic value of 5hmC in grade 4 gliomas with follow-up information and OS. The 5hmC-Seal technique [15], a highly sensitive chemical labeling technique was employed to profile genome-wide 5hmC in tumor samples [26]. The genome-wide 5hmC profiles were used to explore the prognostic biomarkers from genomic features including gene bodies, promoters, and histone modification marks and to evaluate the synergy of these genomic features with clinical parameters and subtypes based on transcriptional characteristics [4]. Additionally, we assessed the crosstalk between 5hmC and gene expression through co-regulation network analysis and identified specific co-regulated modules, in which relevant biological pathways were involved. Our results demonstrate potentially critical roles of 5hmC in regulating transcription as novel prognostic biomarkers for grade 4 gliomas.

## Methods

### Clinical samples

This study was performed under a protocol approved by the Northwestern Institutional Review Board (IRB). All the samples were de-identified before we received them. We obtained fresh-frozen tissue samples of 61 prospectively enrolled adult patients with grade 4 gliomas (≥ 18 years) from the Northwestern Nervous System Tissue Bank (NSTB) at Northwestern University Feinberg School of Medicine, and 9 normal brain samples from the NeuroBiobank at the US National Institutes of Health (https://neurobank.nih.gov) (Table 1). Among the 61 patients with grade 4 gliomas, 63.9% (n = 39) were treatment naïve, 65.6% (n = 40) were of European ancestry, and 83.6% (n = 51) had *IDH1* wild-type (WT) tumors (GBM). The median age of the patients with grade 4 gliomas was 60.0 years (range 46–65 years) and 62.3% (n = 38) were males, and the median age of the normal controls was 55 (range 48–60 years) and 55.6% (n = 5) were males. Diagnosis and grading for the NSTB grade 4 gliomas samples were based on the WHO Classification and Grading System for CNS Tumors Guidelines [1]. Baseline demographic, clinical, pathological, and clinical outcome data, such as age, sex, self-reported race/ethnicity, mutation status, and survival time were retrieved from medical records using our established protocol. The grade 4 gliomas were classified into Neural (N) (n = 4), Proneural (P) (n = 19), Classical (C) (n = 17) and Mesenchymal (M) (n = 21) after the RNA-seq data were processed and normalized as described below using the Simple Glioblastoma Subclassifier [29]. After tissue biopsy, 77.0% (n = 47) patients received standard treatments, including adjuvant radiotherapy (RT) and temozolomide (TMZ) chemotherapy. Thirty-nine (63.9%) patients were deceased with an average OS time of 10.8 (± 9.2) months, after being followed up for 44 months. All samples were randomized for the assays and the technicians were blinded to sample identities. Informed consent was obtained for each participating individual for the NSTB samples.

**Table 1:** Demographics and clinical characteristics of the study participants

Category		Grade 4 glioma		p-value
		*IDH1* mutant (*IDH1*-Mut astrocytoma)	*IDH1* wild type (GBM)	
		n = 10	n = 51	
Age at Diagnosis (yrs)	Mean (sd)	59.5 (12)	39.3 (7)	
	Median (lq,uq)	61.0 (54.5,67.0)	38.0 (34,42.8)	0.00^a^
	No. < 40 yrs	5 (50.0%)	7 (13.7%)	
Sex	Female	3 (30.0%)	20 (39.2%)	0.85^b^
	Male	7 (70.0%)	31 (60.8%)	
Population	African American	1 (10.0%)	1 (2.0%)	
	European American	8 (80.0%)	32 (62.7%)	0.34^b^
	Multiracial	–	5 (9.8%)	
	Unknown	1 (10.0%)	12 (23.5%)	
Molecular Subtype^c^	Classical	1 (10.0%)	16 (31.4%)	
	Mesenchymal	2 (20.0%)	19 (37.3%)	0.03^b^
	Neural	–	4 (7.8%)	
	Proneural	7 (70.0%)	12 (23.5%)	
Pre-Treatment	Treatment Naïve	4 (40.0%)	35 (68.6%)	
	RT	6 (60.0%)	11 (21.6%)	0.08^b^
	TMZ	6 (60.0%)	12 (23.5%)	
Post-Treatment	Treatment Naïve	2 (20.0%)	12 (23.5%)	
	RT	6 (60.0%)	35 (68.6%)	1^b^
	TMZ	8 (80.0%)	34 (66.7%)	
Recurrence Status	Newly-diagnosed	4 (40%)	35 (68.6%)	0.17^b^
	Recurrent	6 (60%)	16 (31.4%)	
Survival Status	Alive	6 (60.0%)	16 (31.4%)	0.17^b^
	Dead	4 (40.0%)	35 (68.6%)	
Overall Survival (months)	Alive—mean (sd)	36.0 (9.6)	18.0 (13.2)	
	Dead—mean (sd)	15.6 (15.6)	10.2 (8.4)	

*IDH1* isocitrate dehydrogenase 1, *RT* radio therapy, *TMZ* temozolomide chemotherapy, *sd* standard deviation, *lq* lower quantile, *uq* upper quantile

^a^Two-tailed Wilcoxon rank sum test

^b^Pearson's Chi-squared test for two-sample proportions

^c^Subtypes predicted based on the expression profiles of core gene signatures

### RNA-seq and bioinformatic processing

Total RNA was isolated using Qiagen RNeasy Kit (Cat #74104) or Trizol, followed by treatment with the Ribo-Zero Gold rRNA Removal Kit (Illumina, Inc., USA) [30]. The cDNA libraries were prepared using the TrueSeq Stranded Total RNA Library Prep Kit (Illumina, Inc., USA) and the next-generation sequencing (NGS) was performed on the Illumina HiSeq 4000 platform (PE50) at the University of Chicago Genomics Facility. Approximately 24 million read pairs were generated from each library. Raw sequencing reads were trimmed and filtered for low-quality bases and reads (quality score ≥ 20 for a minimum of 90% bases) using the FASTX Toolkit (v 0.0.14), followed by alignment to the human genome reference (GRCh37/hg19 without chromosome X, Y and Mitochondria) using the spliced aligner Tophat (v 2.1.0) with the default paired-end mode [31]. Read pairs were concordantly aligned with ≤ 2 mismatches. Aligned read pairs with mapping quality score ≥ 20 were counted for genomic features according to the start and end coordinates derived from the ENCODE-derived [32] annotation files (hg19) using FeatureCounts from Subread (v 1.6.1) with strand information [33, 34].

### Profiling of 5hmC and bioinformatic processing

Genomic DNA was isolated from tissues using the QIAamp DNA Mini Kit (Qiagen, Germany). DNA fragmentation was done by sonication, and quality and quantity examined with standard molecular biology protocols using Qubit (Thermo Fisher, USA). Approximately 50 ng per sample was used to construct the 5hmC-Seal library as we previously described [15, 26, 35, 36], followed by the NGS on the NextSeq 500 platform (PE39) at the University of Chicago Genomics Facility. On average 25 million read counts were obtained for each sample. Robustness of the 5hmC-Seal technique, including reproducibility and comparison with the “gold standard” TAB-seq has been previously described [15, 26, 36, 37].

The raw 5hmC-Seal data were summarized using the pipelines that we previously described [26, 35, 38]. Briefly, raw sequencing reads were trimmed and filtered for low-quality bases using the FASTX Toolkit (v 0.0.14), followed by alignment to hg19 using Bowtie2 (v 2.2.6) with the end-to-end alignment mode [39]. Read pairs were concordantly aligned with fragment length ≤ 500 bp and with up to one ambiguous base and up to four mismatched bases per 100 bp. Aligned read pairs were then sorted, indexed and deduplicated using Picard (v 2.6.0). Alignments with mapping quality score ≥ 10 were counted for gene bodies, and histone modifications [35].

### Differential analysis between grade 4 gliomas and normal brain tissues

**Fig. 1:**
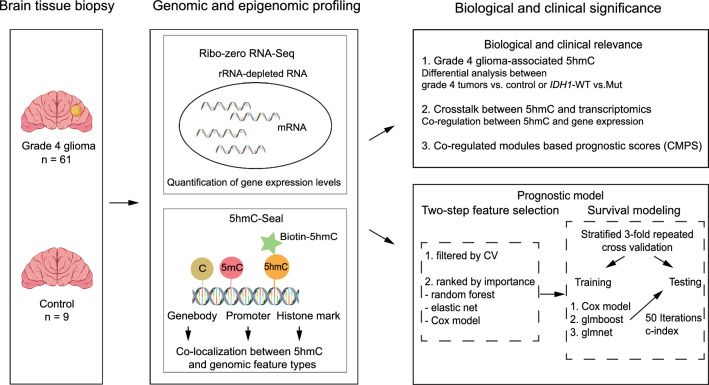
Study population and an overview of workflow. The 5hmC and transcriptomic profiles obtained in tissue samples from a set of patients with grade 4 gliomas (n = 61) and normal controls (n = 9) are investigated for grade 4 gliomas-associated expression and 5hmC, as well as co-regulated modules by interaction type (i.e., mRNA–mRNA, 5hmC–5hmC, 5hmC–mRNA). The 5hmC-derived prognostic scores based on co-regulated modules are explored for patient overall survival using machine learning algorithms, such as elastic net and random forest, and the Cox proportional hazards model. 5hmC: 5-hydroxymethylcytosine, C-index: Harrell’s C-index (the concordance index)

Differential 5hmC and transcription was analyzed using the DESeq2 (v 1.30.1) (Fig. 1) [40] for various genomic features with at least 5 read counts across 80% of samples. Multivariable logistic regression models adjusted for gender and 10-yrs age groups were used to identify features differentially modified or expressed at 5% false discovery rate (FDR) between grade 4 gliomas and normal brain tissues, as well as between *IDH1* mutant astrocytoma (Mut) and *IDH1*-wild type (GBM) grade 4 gliomas. For downstream analysis, the raw sequencing data were transformed using the variance stabilizing method [40]. All statistical analysis was performed under the R Statistical Computing Environment (v 4.0.3) [41].

To determine whether the associations between gene expression—mRNA and local/*cis*-acting gene body_5hmC_ and promoter_5hmC_ altered in grade 4 gliomas as compared to normal brain tissues or in *IDH1*-WT as compared to *IDH1*-Mut, an equal number of samples (n = 9) were randomly selected from each group (i.e., Control, *IDH1*-WT [GBM], and *IDH1*-Mut astrocytoma). Pearson correlation coefficients (*R*) were computed between gene expression and 5hmC levels within each group of samples. Correlations are categorized into positive (*R* > 0), negative (*R* < 0), and strong correlations (|*R*|≥ 0.5) based on the *R* values. The over-representation analysis (ORA) was conducted to identify molecular pathways (≥ 10 component genes and hypergeometric test adjusted p-value < 0.05) from the Kyoto Encyclopedia of Genes and Genomes (KEGG) associated with genes with altered correlations [42].

### Co-regulation analysis between gene expression and 5hmC

Genomic features were first filtered based on the p-values (0.01 or the default p-value of 0.1), which were calculated by modeling the variance of genes as an inverse gamma distribution [43]. To identify co-regulated 5hmC–5hmC, mRNA–mRNA, or 5hmC–mRNA modules, the modified weighted gene co-expression network analysis (WGCNA) with improved soft-threshold selection was conducted using the CEMiTool with default parameters [43, 44]. Once the co-regulated modules were identified, ORA was conducted to assess whether these co-regulated modules were enriched with any KEGG pathways and Gene Ontology (GO) biological processes (≥ 5 component genes and hypergeometric test adjusted p-value < 0.05) [42, 45]. The Gene Set Enrichment Analysis (GSEA) [43, 46] was also conducted to evaluate the association between these co-regulated modules and clinical classes (e.g., by *IDH1* mutation) based on the normalized enrichment score (NES) and adjusted empirical p-values. Furthermore, protein–protein interactions from Reactome were integrated with the co-regulated modules to identify master regulators/players for each co-regulation type (e.g., 5hmC–5hmC) [43, 46, 47].

### Prognostic significance of co-regulated modules

Cox models were developed for individual modules and the combined modules within each co-regulation type to assess whether their prognostic significance. Specifically, univariate Cox models were first constructed to evaluate the association between the eigengene values (i.e., the first principal component) of individual modules and OS. Within each interaction type (e.g., mRNA–mRNA), multivariable Cox models were further constructed using all modules. A weighted Co-regulated Module-based Prognostic Score (CMPS) was then calculated for each sample. Samples were categorized into low- and high-risk groups based on the median of CMPS. The Kaplan-Meir (KM) models were built to evaluate differential OS survival. Multivariable Cox models adjusting for covariates such as age, gender, or *IDH1* mutation were also developed to evaluate the association between CMPS (numeric) and survival probability. The time-dependent Receiver Operating Characteristic (ROC) curve and the Area under the ROC Curve (AUROC) were plotted to compare the performances between CMPS and conventional prognostic factors, such as *IDH1* mutation.

### Developing integrative prognostic models for grade 4 gliomas

To identify prognostic signatures that are independent of conventional prognostic factors such as *IDH1* mutation status, the 5hmC-, mRNA-based prognostic models were developed using a two-step procedure for each genomic feature type (i.e., promoter_5hmC_, H3K27ac_5hmC_ and gene body_5hmC_, and mRNA, separately (Fig. 1). In Step 1, for more efficient modeling and feature selection, we selected a list of candidate features by filtering out those with less variation (i.e., less informative), based on coefficient of variance (CV) < upper quartile (CV) across grade 4 gliomas. In Step 2, the candidate features from step 1 were further ranked and selected to build a final prognostic model by applying machine learning algorithms or statistical models, such as random forest, univariate Cox model, and generalized linear model via penalized maximum likelihood. Specifically, genomic features will be ranked by importance using the abovementioned methods, separately. The top 10, 20, 30, 40 and 50 features that were present in each feature selection method were retained and evaluated using 50 repeated threefold stratified cross-validation under survival prediction models such as univariate Cox model, gradient boosted generalized linear survival learner (glmboost), and the generalized linear survival learner with elastic net regularization (glmnet). In addition, gender (categorical), age (continuous), and *IDH1* mutation (categorical) were added into the survival prediction models [48, 49]. The Harrell’s concordance index (i.e., c-index, a weighted average of time-specific AUCs) and 95% confidence intervals (CI) of the testing set within each iteration were used as the performance metrics to select the best models [50]. Patients with grade 4 gliomas were further classified into low- and high- risk groups based on the predicted risk scores obtained from the best model by summing up the products of coefficients and final variables. The KM survival analysis was conducted to evaluate differential OS between the low- and high-risk groups, as determined by the log-rank test.

## Results

### Genome-wide distributions indicate re-wiring of 5hmC and gene expression relationships in grade 4 gliomas

**Fig. 2:**
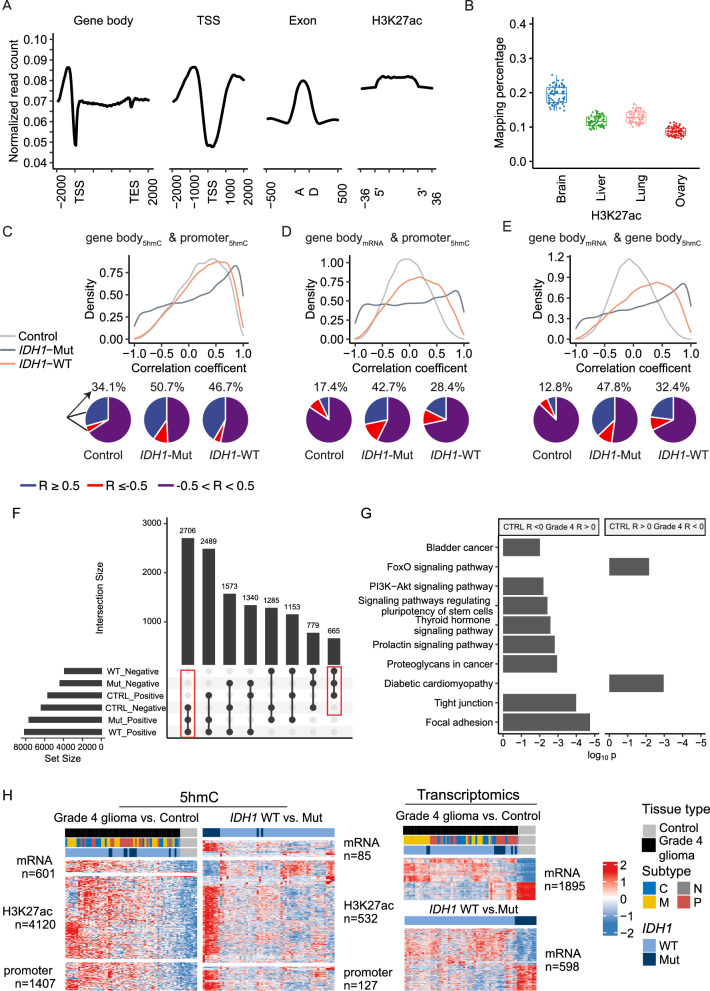
Genome-wide 5hmC and transcriptomic landscapes in grade 4 gliomas and normal brain tissues. **A** The 5hmC modifications are distinctly distributed across various genomic features. The read counts are normalized to per million counts. TSS, transcription start site; TES, transcription end site; A, splicing acceptor site; D, splicing donor site. **B** The 5hmC profiles in grade 4 gliomas and normal brain tissues are featured with tissue-specificity with a higher co-localization proportion of brain-derived enhancer markers (H3K27ac), compared with other organs. Distinct correlation patterns are observed for grade 4 gliomas and normal controls between: **C** local 5hmC features (i.e., promoters and gene bodies); **D** local 5hmC promoter and gene expression; and **E** local gene body 5hmC and gene expression. **F** Dissection of genes with re-wired 5hmC and transcription relationships in grade 4 gliomas. **G** Shown are KEGG pathways enriched among genes with re-wired 5hmC and transcription relationships in grade 4 gliomas. **H** The heatmaps show differential 5hmC features (FDR < 0.05) and mRNA transcripts (FDR < 0.05) detected between patients with grade 4 gliomas and normal controls, or between *IDH1* (encoding isocitrate dehydrogenase 1) wild-type (WT) tumors (GBM) and mutants (grade 4, *IDH* mutant astrocytoma) in grade 4 gliomas. FDR, false discovery rate; Molecular subtypes are annotated as C (classical), N (neural), M (mesenchymal), and P (proneural)

The distributions of 5hmC were compared across various genomic features. Similar to our observations in the cfDNA samples from gliomas [35], the 5hmC-Seal reads profiled in these tissues were more abundant in gene bodies and exonic regions relative to their flanking regions and depleted at the promoter regions (Fig. 2A). Notably, the distribution of 5hmC-contatining reads from both grade 4 gliomas and normal controls showed more co-localization with brain-derived enhancer markers, e.g., H3K27ac loci, when compared with other tissue types (pair-wise one-tailed z-test p < 0.01), including liver, lung, and ovary from the Roadmap Epigenomics Project (Fig. 2B), consistent with the tissue-specificity and putative roles of 5hmC in gene activation.

In contrast to the well-established inverse correlation between promoter methylation and gene expression, the relationship between 5hmC modifications (promoter_5hmC_ or gene body_5hmC_) and gene expression (mRNA) appeared to be bidirectional, and significantly more positive correlations (*R* > 0) were observed in *IDH1*-WT tumors as compared to normal controls or *IDH1*-Mut tumors (one-tailed z-test p < 0.001) (Fig. 2C–E). For example, in normal controls, 47.4% of genes showed positive correlations between promoter_5hmC_ and mRNA of its host gene, while that proportion increased to 60.6% and 55.0% in *IDH1*-WT and *IDH1*-Mut tumors, respectively. Notably, the proportions of those strong correlations (|*R*| ≥ 0.5) between promoter_5hmC_ and mRNA remained to be significantly higher in *IDH1*-Mut tumors (42.7%) as compared to *IDH1*-WT tumors (28.4%) or normal controls (17.4%) (Fig. 2D). The same trend was also observed between promoter_5hmC_ and gene body_5hmC_ (Fig. 2C) and between gene body_5hmC_ and mRNA (Fig. 2E). Of note, ~ 22.5% of the genes showed negative correlations between gene body_5hmC_ and host-gene mRNA in normal controls but positive associations in grade 4 gliomas (i.e., re-wired 5hmC-expression features) were found to be significantly associated with pathways such as focal adhesion, tight junction and PI3K-Akt signaling pathway (hypergeometric test p < 0.01 and gene count ≥ 10) (Fig. 2F–G).

### Differentially modified/expressed genomic features associated with grade 4 gliomas

Differential analyses of 5hmC modifications identified 601 gene bodies, 1407 promoter regions, and 4120 H3K27ac loci between patients with grade 4 gliomas and normal controls at 5% FDR and fold change > 50% (Fig. 2H, Additional file 4: Table S1). In contrast, 1895 mRNAs were found to be differentially expressed between patients with grade 4 gliomas and normal controls at 5% FDR and fold change > 4 (Fig. 2H, Additional file 4: Table S1). Additionally, differential analyses between *IDH1*-WT and *IDH1*-Mut tumors (Fig. 2H, Additional file 5: Table S2) showed 85 gene bodies, 127 promoter regions, and 532 H3K27ac loci with differential 5hmC modification levels, while 598 mRNAs showed differential expression associated with the mutation status (Fig. 2H, Additional file 5: Table S2), as demonstrated by hierarchical clustering as well (Fig. 2H). Of note, KEGG enrichment analysis of genes with both higher 5hmC modifications and gene expressions in grade 4 gliomas suggested their enrichments with molecular pathways involved in transcriptional mis-regulation in cancer.

Interestingly, we observed a higher proportion of differential features (e.g., gene bodies) in expression levels compared to corresponding 5hmC modifications, independent of the fold change thresholds (Additional file 1: Fig. S1A). For example, approximately 60% of mRNAs were differentially expressed (FDR < 0.05) while less than 25% of mRNAs showed differential hydroxymethylation (FDR < 0.05) between patients with grade 4 gliomas and normal controls, and only less than 25% mRNAs were both differentially expressed and hydroxymethylated (Additional file 1: Fig. S1A). In addition, the majority of differential 5hmC features (> 95%) showed higher modification levels in grade 4 gliomas compared to normal controls (Additional file 1: Fig. S1A). In contrast, gene expression showed a more even proportion of both up- and down-regulation in grade 4 gliomas compared to normal controls (Additional file 1: Fig. S1A).

### Co-regulation between gene expression and local 5hmC

**Fig. 3:**
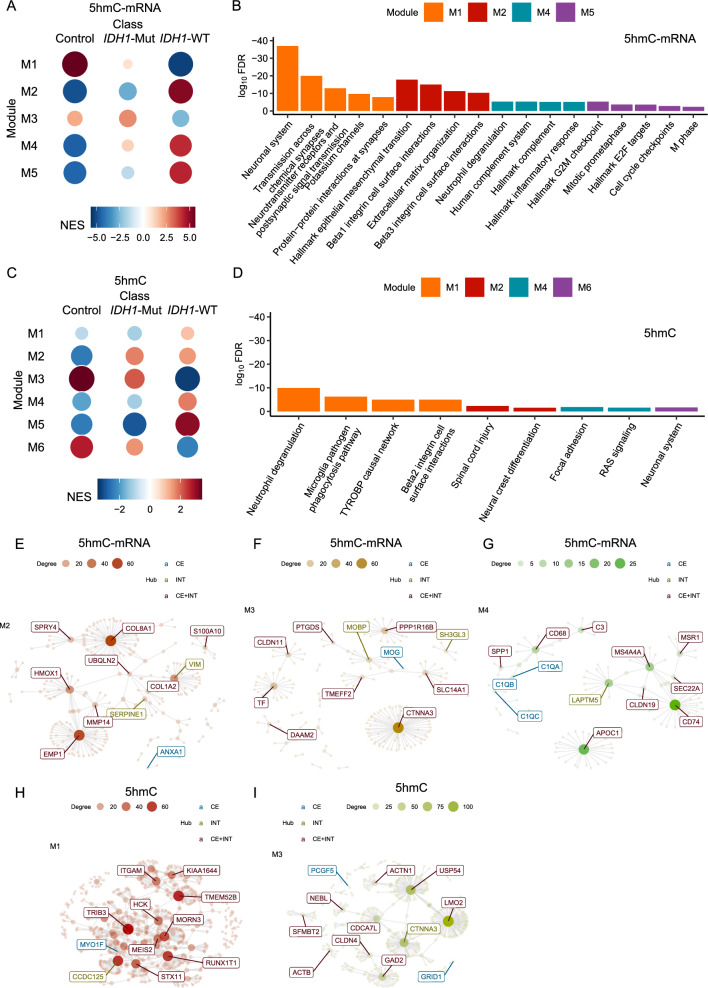
Integrative analysis of co-regulated modules in grade 4 gliomas and normal brain tissues. Co-regulated 5hmC and transcription modules are derived from the modified weighted gene co-expression network analysis (WGCNA) in grade 4 gliomas and normal brain tissue samples, separately. **A** Enriched co-regulated 5hmC–mRNA modules are identified in normal controls, *IDH1* (encoding isocitrate dehydrogenase 1) mutants (*IDH1-Mut* astrocytoma), and *IDH1* wild-type (*IDH1-WT* GBM) tumors. **B** Top five enriched GSEA gene sets associated with 5hmC–mRNA co-regulated modules (FDR < 0.05 and gene count > 5) are shown. **C** Enriched co-regulated 5hmC–5hmC modules are detected in normal controls, *IDH1*-Mut, and *IDH1*-WT tumors. **D** Top five enriched GSEA gene sets associated with 5hmC–5hmC co-regulated modules (FDR < 0.05 and gene count > 5) are shown. **E**–**G** Network hubs of co-regulated 5hmC–mRNA modules are shown for respective modules. **H**, **I** Network hubs of co-regulated 5hmC modules are shown for respective modules. *FDR* false discovery rate, *GSEA* Gene Set Enrichment Analysis. CE denotes co-expression/regulation hubs; INT denotes protein–protein interaction hubs; CE + INT denotes co-expression/regulation and protein–protein interaction hubs

The co-regulation analysis was conducted on the 12,975 genes with both 5hmC and gene expression data. Five co-regulated 5hmC–mRNA modules were identified and were associated significantly with normal controls and *IDH1*-WT (FDR < 0.05), while two of them (module M2 and M3) were significantly associated with *IDH1*-Mut (Fig. 3A, Table 2). Module M2 (NES = 5.12, FDR < 0.001), module M4 (NES = 4.18, FDR < 0.001), and module M5 (NES = 4.3, FDR < 0.001) that contained expression data of 68, 40, and 38 genes, respectively, were significantly up-regulated in patients with *IDH1* wild-type alleles (Fig. 3A, Table 2 & Additional file 6: Table S3), while in *IDH1*-Mut, module M2 was significantly down-regulated (NES = − 2.78, FDR < 0.001). Interestingly, module M2 was significantly enriched with pathways relevant to epithelia mesenchymal transition, integrin-related pathways, and extracellular matrix organization, while module M4 is primarily enriched with inflammatory response such as neutrophil degranulation, and module M5 is primarily enriched with cell-cycle related pathways (Fig. 3B, Additional file 7: Table S4). Module M1 comprised of 366 mRNAs and *EGFR*’s 5hmC level, was significantly down-regulated in *IDH1*-WT tumors ([NES = − 5.23, FDR < 0.001) and up-regulated in normal controls (NES = 5.59, FDR < 0.001) (Fig. 3A, Additional file 6: Table S3). Notably, module M1 was found to be enriched with pathways involved in synapses and neuronal system (Fig. 3B, Additional file 7: Table S4). Of note, the pathways underlying the co-expression of genes (mRNA modules) were similar compared to those in the 5hmC–mRNA modules. For example, pathways such as “Neuronal system” and “Transmission across chemical synapses” were identified in M1 from both “only mRNA” (Additional file 1: Fig. S1B, C) and “5hmC–mRNA” analysis (Fig. 3A, B), which however were different from pathways involved in “only 5hmC modules” (Fig. 3C, D).

**Table 2:** Enrichment of co-regulated 5hmC and transcriptomic modules by phenotype

Module Type	Module	Number of Features	Control NES (FDR)	GBM NES (FDR)	*IDH1*-Mut astrocytoma NES (FDR)
5hmC–mRNA	M1	367	5.59 (0)	− 5.23 (0)	0.87 (0.88)
	M2	68	− 4.91 (0)	5.12 (0)	− 2.78 (0)
	M3	66	2.13 (0)	− 2.53 (0)	2.65 (0)
	M4	40	− 4.59 (0)	4.18 (0)	1.31 (0.113)
	M5	38	− 4.48 (0)	4.3 (0)	− 1.49 (0.063)
5hmC only	M1	213	− 0.92 (0.771)	0.99 (0.6)	− 1.15 (0.155)
	M2	181	− 2.43 (0)	1.49 (0.005)	1.72 (0)
	M3	106	3.42 (0)	− 3.22 (0)	2.11 (0)
	M4	97	− 1.85 (0)	1.77 (0.001)	− 1.19 (0.155)
	M5	77	− 2.41 (0)	3.07 (0)	− 2.91 (0)
	M6	54	2.69 (0)	− 2.34 (0)	1.54 (0.032)
Transcripts only	M1	463	6.48 (0)	− 6.54 (0)	1.29 (0.009)
	M2	92	− 4.49 (0)	4.93 (0)	− 2.63 (0)
	M3	89	− 5.17 (0)	4.74 (0)	1 (0.445)
	M4	53	− 4.65 (0)	4.49 (0)	− 1.83 (0.001)
	M5	40	− 2.63 (0)	− 2.04 (0.001)	2.45 (0)

*NES* normalized enrichment score, *FDR* false discovery rate for the hypergeometric test, *IDH1* isocitrate dehydrogenase 1

Moreover, the integrative analysis of protein–protein interactions and co-regulated 5hmC–mRNA modules highlighted important genes as the network hubs, such as VIM (encoding vimentin) and SERPINE1 (encoding serpin family E member 1) in module M2 (Fig. 3E), MOBP (encoding myelin associated oligodendrocyte basic protein) and SH3GL3 (encoding SH3 domain containing GRB2 like 3) in module M3 (Fig. 3F), as well as LAPTM5 (encoding lysosomal protein transmembrane 5) in module M4 (Fig. 3G, Additional file 2: Fig. S2A-B). Of note, these genes have been implicated in grade 4 glioma biology. For example, LAPTM5 can suppress invasion and sensitize TMZ treatment [51]. SERPINE1 is a well-known mesenchymal signature related with cancer progression and poor prognosis in patients with grade 4 gliomas [52].

### Co-regulation of 5hmC modifications over gene bodies

Because the co-regulation between 5hmC modification and gene expression primarily reflected the co-expression of genes (Fig. 3A, C, Additional file 1: Fig. S1B-C, Additional file 6: Table S3), we further investigated the co-regulation of 5hmC modifications over gene bodies. Six co-regulated 5hmC modules were identified. Among them, five were associated significantly with normal controls and *IDH1*-WT tumors (FDR < 0.05), and four were significantly associated with *IDH1*-Mut (Fig. 3C, Table 2). Module M2, M4, and M5 that contained 5hmC data of 181, 97, and 77 genes, respectively, were significantly up-modified in patients with *IDH1*-WT tumors, while module M3 and module M6 were down-modified (Fig. 3C, Additional file 6: Table S3). In tumors with *IDH1* mutations, module M2, M3, and M6 were significantly up-modified, while module M5 was down-modified. Though module M1 was enriched with genes involved in integrin-related pathways, TYROBP casual network, and neutrophil degranulation, no significant associations were observed between module M1 and normal controls and tumors regardless of *IDH1* mutation status (Fig. 3D, Additional file 6: Table S3). Despite no pathways overrepresented in module M3 and module M5, module M2 and M6 were found to be enriched with genes involved in neuronal system, while module M4 was primarily enriched with Ras signaling (Fig. 3D, Additional file 7: Table S4). The integrative analysis of protein–protein interactions and co-regulated 5hmC modules highlighted cancer relevant genes as the network hubs (Fig. 3H, I, Additional file 2: Fig. S2D-F), including CCDC125 (encoding coiled-coil domain containing 125) in module M1 (Fig. 3H) and CTNNA3 (encoding catenin alpha 3) in module M3 (Fig. 3I), distinct from what was observed from the mRNA–5hmC co-regulation analysis.

### Prognostic significance of co-regulated modules

**Fig. 4:**
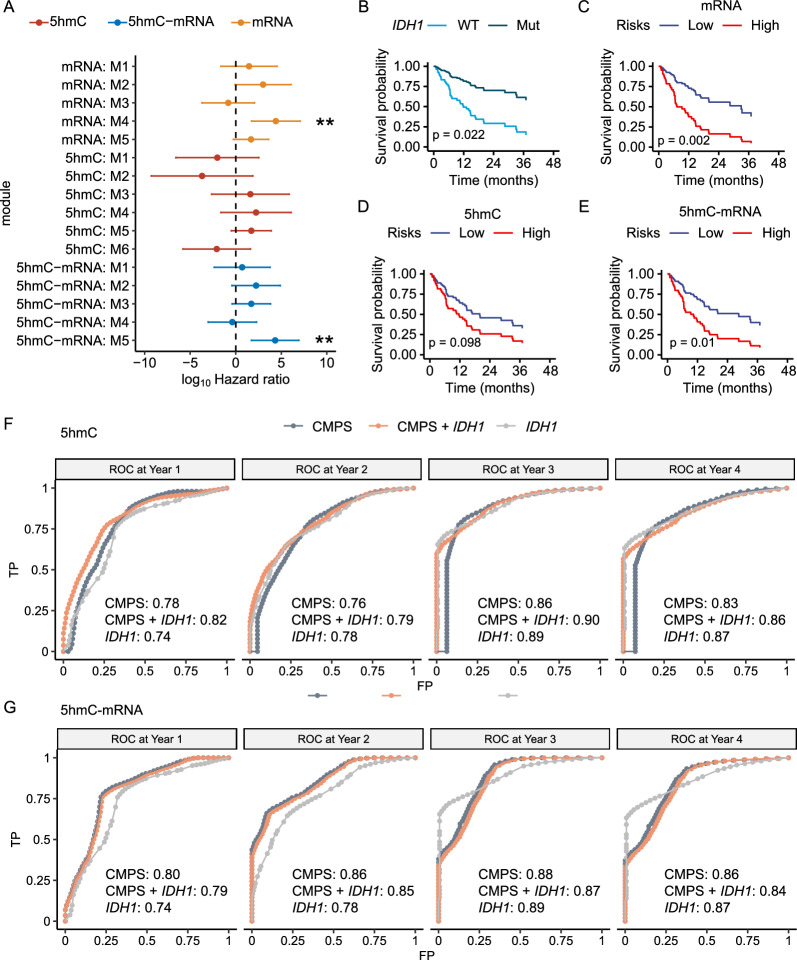
Prognostic significances of co-regulated modules and prediction models. **A** Forest plots showing hazard ratios (HR) of different co-regulated modules. Kaplan–Meier survival curves demonstrating significant differences between **B** *IDH1*-WT (GBM) and *IDH1*-Mut astrocytoma tumors; **C** low- and high-risk groups categorized based on mRNA–mRNA co-regulated modules derived CMPS; **D** low- and high-risk groups categorized based on 5hmC–5hmC co-regulated modules derived CMPS; **E** low- and high-risk groups categorized based on 5hmC–mRNA co-regulated modules derived CMPS. **F** Time-dependent ROC curves for patients’ survival with AUC measures evaluated using 5hmC co-regulated modules derived CMPS. **G** ROC curves for patients’ survival with AUC measures evaluated using 5hmC–mRNA co-regulated modules derived CMPS

In the 61 grade 4 glioma samples with complete survival and clinical information, age at diagnosis (p < 0.05), gender (p < 0.01), *IDH1* mutation status (p < 0.05), post-radiotherapy treatment (p < 0.001), and post-TMZ treatment (p < 0.0001) after sample collection were found to be potential prognostic factors. Module-wise, co-regulated mRNA module M4 ([log_10_(HR) = 4.40 (1.73–7.07)]) and 5hmC–mRNA module M5 ([log_10_(HR) = 4.33 (1.73–6.93)]) were significantly associated with survival, and both modules are significantly associated with *IDH1*-WT tumors (Figs. 3A, C, 4A). Two groups’ Kaplan–Meier survival curves showed that samples with higher mRNA-, and 5hmC–mRNA-derived CMPS had significantly shorter survival time, with 5hmC–mRNA-derived CMPS performing comparably equal or better than conventional prognostic factor *IDH1* mutation status in predicting survival probability (Fig. 4B–E). Time-dependent ROC and AUC analyses showed that 5hmC-derived CMPS (AUC = 0.78) discriminated patients with higher risk at Year 1 with better accuracy than *IDH1* (AUC = 0.74); when combined with *IDH1*, 5hmC-derived CMPS discriminated patients with higher risk at all years with comparable or even better accuracy than *IDH1* (Fig. 4F). As for 5hmC–mRNA-derived CMPS, its discriminatory performance peaked at Year 3, and outperformed *IDH1* at both Year1 and Year2 (Fig. 4G). Of note, within those *IDH1*-WT tumors, the time-dependent ROC and AUC analyses showed that the 5hmC-derived CMPS (AUC = 0.81) discriminated patients with higher risk at Year 1, and its discriminatory performance peaked at Year 3 (Additional file 3: Fig. S3A). As for the 5hmC–mRNA-derived CMPS, its discriminatory performance was found to peak at Year 2 (AUC = 0.79), suggesting that the 5hmC-based signatures would be identifying high risk cases in the *IDH1*-WT cohort, independent from the mutation status (Additional file 3: Fig. S3B).

### Prognostic models for OS

**Fig. 5:**
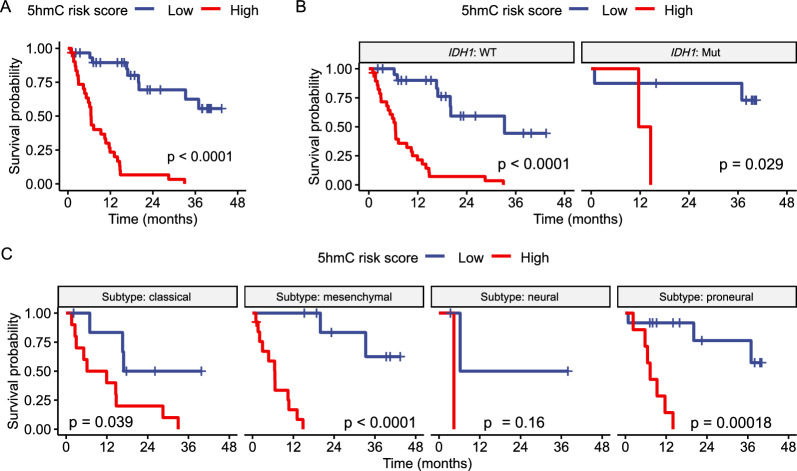
Performance of prognostic models. **A** Kaplan–Meier survival curves demonstrating significant differences between low-and high-risk groups categorized based on the predictive risk scores from the best-performed prediction model. Kaplan–Meier survival curves demonstrating significant differences between low-and high-risk groups categorized based on the predictive risk scores from the best-performed prediction model in **B** *IDH1*-WT (GBM) and *IDH1*-Mut astrocytoma tumors; **C** molecular subtypes. CMPS denotes Co-regulated Modules based Prognostic Score

To identify prognostic signatures without the influence of prior prognostic information (e.g., association with *IDH1*), The 5hmC modification levels over annotated mRNAs, promoters, brain-derived H3K27ac histone markers as well as gene expression were selected, modeled, and evaluated separately and integratedly for association with patient survival under repeated threefold cross validation. The prognostic model comprised of the 5hmC modification levels of 30 promoters (Additional file 3: Fig. S3D) alone achieved the best performance in predicting OS in the testing datasets (average c-index = 74%, 95% CI, 60–87%), outperforming other genomic features or conventional prognostic factors (Table 3). However, when combined with *IDH1* mutation status or other covariates such as age and gender, the average c-index of this 5hmC-based model could be slightly reduced to 72% (95% CI, 59–86%), suggesting its independence from other prognostic factors. We further retrieved the predicted risk scores derived from the best-performed glmboost prognostic model based on the 5hmC modification levels of the 30 promoters as described earlier. Of note, patients with grade 4 gliomas with higher predicted risk scores (relative to median) were associated significantly with shorter survival regardless of the *IDH1* mutation status (p < 0.05) (Fig. 5A. B). Even within the molecular subtypes such as classical, mesenchymal and proneural, patients with grade 4 gliomas can be further categorized into two groups with significant survival difference, with higher predicted risk scores associated significantly with shorter survival (Fig. 5C).

**Table 3:** Performance of the prognostic models comprised of various 5hmC and transcriptomic features

Data	5hmC			Transcriptome	5hmC-Transcriptome
Feature type	Promoter	H3K27ac	Gene body	mRNA	Integrated
Model	Glmboost	Glmnet	Glmboost	Glmboost	Glmboost
Feature Selection Method	rf	uc	rf	rf	rf
Feature Number	30	20	20	10	30
*IDH1*	0.57 (0.47–0.67)				
Age + Gender + *IDH1*	0.68 (0.53–0.83)				
PS	0.74 (0.60–0.87)	0.72 (0.58–0.86)	0.69 (0.53–0.85)	0.70 (0.56–0.85)	0.74 (0.60–0.88)
Age + Gender + PS	0.74 (0.61–0.87)	0.70 (0.58–0.83)	0.70 (0.57–0.83)	0.72 (0.58–0.85)	0.71 (0.57–0.86)
Age + Gender + *IDH1* + PS	0.72 (0.59–0.86)	0.69 (0.55–0.84)	0.68 (0.54–0.83)	0.72 (0.59–0.85)	0.72 (0.58–0.86)

Average Harrell’s concordance index (c-index) and 95% confidence intervals (CI) of the testing sets are shown for each model

*glmboost* gradient boosted generalized linear survival learner, *glmnet* generalized linear survival learner with the elastic net regularization, *rf* random forest, *uc* univariate Cox proportional hazards model, *PS* prognostic signatures based on 5hmC, transcriptome, or integrated, *IDH1* isocitrate dehydrogenase 1

## Discussion

In this study, we primarily evaluated the prognostic significance of 5hmC in grade 4 glioma tissues. We identified phenotype associated co-regulated 5hmC–5hmC or 5hmC–mRNA modules that could provide novel insights into heterogeneity and tumorigenesis as well as potential crosstalk between 5hmC and transcription levels in grade 4 gliomas. Specifically, we developed prognostic models for grade 4 gliomas from the 5hmC/transcription levels of a variety of genomic features and evaluated their performance in predicting patient survival. For example, in our tested grade 4 gliomas, *IDH1* mutation status, the well-known prognostic factor, predicted patient survival at an average c-index of 57% (95% CI, 47–67%) in the testing data sets. In contrast, our best-performing 5hmC model predicted patient survival at a much higher c-index of 74% (95% CI, 60–87%), representing ~ 29.8% improvement. In addition, the predicted risk scores computed based on the best-performing 5hmC model could stratify patients with grade 4 gliomas into two groups with significant survival differences independent of *IDH1* mutations or certain molecular types, suggesting that the 5hmC model captured the molecular characteristics of tumors that are independent of *IDH1* mutation status and gene expression-based molecular subtypes. However, future studies with larger sample size and independent validation are needed to establish the clinical validity of this model.

To provide more insights in the epigenetic contribution to grade 4 gliomas, we further evaluated the connection between 5hmC modification and gene expression. A closer look at the co-regulated modules and their associations with key biological pathways shed some light into to the crosstalk between 5hmC and gene expression as well as molecular heterogeneity in patients with grade 4 gliomas. For example, the 5hmC modification levels of *EGFR* were found to be co-regulated with the expression of genes implicated in neuronal system and synaptic signal transmission. Given that *EGFR* is a key driver of tumorigenesis and has important neurotrophic functions, it is likely to be a crosstalk hub between 5hmC and gene dysregulation in tumors [53]. In addition, enrichment analysis identified shared pathways that were overrepresented in co-regulated 5hmC–5hmC/5hmC–mRNA/mRNA–mRNA modules such as pathways involved in neuronal system, neutrophil degranulation, and integrin cell interactions. Of note, integrated protein–protein interaction and co-regulation analyses identified different network hubs for co-regulated 5hmC–5hmC or 5hmC–mRNA or mRNA–mRNA modules. The co-regulated 5hmC–mRNA modules were primarily dictated by co-expressed mRNAs, with hubs genes previously known to be involved in glioma tumorigenesis. Network analysis of co-regulated 5hmC–5hmC modules revealed novel hub genes whose function or in particular their hydroxymethylation have been less investigated in grade 4 gliomas. For example, MYO1F (myosin 1F), a network hub in co-regulated 5hmC–5hmC module M1, was critical for neutrophil trafficking [54]. MSI2 (RNA-binding protein Musashi-2), a network hub in co-regulated 5hmC–5hmC module M2, has been implicated in tumorigenesis and progression in certain human cancers.

Technically, the 5hmC-Seal approach has showed value for cancer biomarker discovery in gliomas as well as other human cancers from tissue samples or circulating materials [26, 35, 38]. Our findings utilizing tissue samples from patients with grade 4 gliomas further supported the tissue or tumor relevance of blood-derived 5hmC profiles that we recently reported [35]. However, there are several limitations that could be addressed in future studies. First, the current study is limited of sample size and lacks independent validation dataset, future studies with larger sample size and more comprehensive pathological (e.g., tumor cellularity), demographic and clinical information will help address problems such as the potential selection bias or suboptimal classification for our samples as well as population/ethnicity disparities. Second, the current study only focused on the co-regulation over genic regions given the functional relevance of genic regions are better annotated and established, it would be interesting to extend the co-regulation between gene expression and 5hmC modification to other genomic regions such as enhancer markers. Finally, future development needs to consider the prognostic significance of integrated model of 5hmC modification, transcriptomic abundance of noncoding transcripts, or other types of omics data. Nonetheless, our findings from the current study warrant further investigations using this novel approach in brain cancer.

## Conclusions

In conclusion, we have developed prognostic models for patients with grade 4 gliomas and investigated the crosstalk between 5hmC and gene expression through an integrative co-regulation and network analysis. The 5hmC-based prognostic model could offer a robust tool to predict survival in patients with grade 4 gliomas, potentially outperforming existing prognostic factors such as *IDH1* mutations. The crosstalk between 5hmC and gene expression revealed another layer of complexity underlying the molecular heterogeneity in grade 4 gliomas, offering opportunities for identifying novel therapeutic targets as well.

### Supplementary Information

**Additional file 1**. Fig. S1. (A) The percentages of genes with differential 5hmC modification and expression under different fold change cutoffs. BG denotes background gene; FDR denotes mRNA/lncRNA with FDR <0.05; Up: up-modified/expressed; down: down-modified/expressed. (B) Enriched co-regulated mRNA-mRNA (gene expression) modules are detected in normal controls, *IDH1*-Mut, and *IDH1*-WT tumors. (C) Top five enriched KEGG pathways associated with mRNA-mRNA co-regulated modules (FDR < 0.05 and gene count > 5) are shown.

**Additional file 2**. Fig. S2. (A) Network hubs of co-regulated 5hmC-mRNA module M1. (B) Network hubs of co-regulated 5hmC-mRNA module M5. (C-F) Network hubs of co-regulated 5hmC-5hmC module. (G-K) Network hubs of co-regulated mRNA modules. CE denotes co-expression/regulation hubs; INT denotes protein-protein interaction hubs; CE+INT denotes co-expression/regulation and protein-protein interaction hubs.

**Additional file 3**. Fig. S3. Time-dependent ROC curves for *IDH1*-WT tumor patients’ survival with AUC measures evaluated using (A) 5hmC-5hmC co-regulated modules derived CMPS; (B) 5hmC-mRNA co-regulated modules derived CMPS; (C) mRNA-mRNA co-regulated modules derived CMPS. (D) Forest plots showing hazard ratios (HR) of the 30 promoters in the best-performed model. HR > 1 indicates increased survival risk per unit change in the 5hmC value; HR < 1 indicates decreased survival risk per unit change in the 5hmC value.

**Additional file 4**. Table S1. Differentially modified 5hmC features and differentially expressed mRNAs between grade 4 gliomas and normal controls.

**Additional file 5**. Table S2. Differentially modified 5hmC features and differentially expressed mRNAs between *IDH1-*WT (GBM) and *IDH1*-Mut astrocytoma tumors.

**Additional file 6**. Table S3. Component features in each module.

**Additional file 7**. Table S4. Pathways associated with each module.

## Data Availability

Due to the patients’ confidentiality, the raw sequencing data are not shared on a public server. Anyone interested in re-analysis of these data is welcome to contact the corresponding authors. Individual-level processed RNA-seq data and 5hmC-Seal data have been deposited into the NCBI Gene Expression Omnibus database (GSE196533).

## Abbreviations

WHO: World health organization
GBM: Glioblastoma
CNS: Central nervous system
OS: Overall survival
IDH1: Isocitrate dehydrogenase 1
EGFR: Epidermal growth factor receptor
ncRNA: Non-coding RNA
MGMT: O-6-methylguanine-DNA methyltransferase
5mC: 5-Methylcytsoines
5hmC: 5-Hydroxymethylcytosines
IRB: Institutional review board
NSTB: Nervous system tissue bank
WT: Wild-type
Mut: Mutant
RT: Radiotherapy
TMZ: Temozolomide
FDR: False discovery rate
ORA: Over-representation analysis
KEGG: Kyoto encyclopedia of genes and genomics
WGCNA: Weighted gene co-expression network analysis
GO: Gene ontology
GSEA: Gene set enrichment analysis
NES: Normalized enrichment score
CMPS: Co-regulated module-based prognostic score
KM: Kaplan-Meir
ROC: Receiver operating characteristic
AUROC: Area under the ROC curve
CV: Coefficient of variance
C-index: Harrell's’ concordance index
CI: Confidence intervals
