# Supplementary figures and images for: Dissection of transcriptomic and epigenetic heterogeneity of grade 4 gliomas: implications for prognosis
Source: Acta Neuropathol Commun. 2023 Aug 14;11:133. doi: 10.1186/s40478-023-01619-5 (PMC10426201; doi:10.1186/s40478-023-01619-5)

A

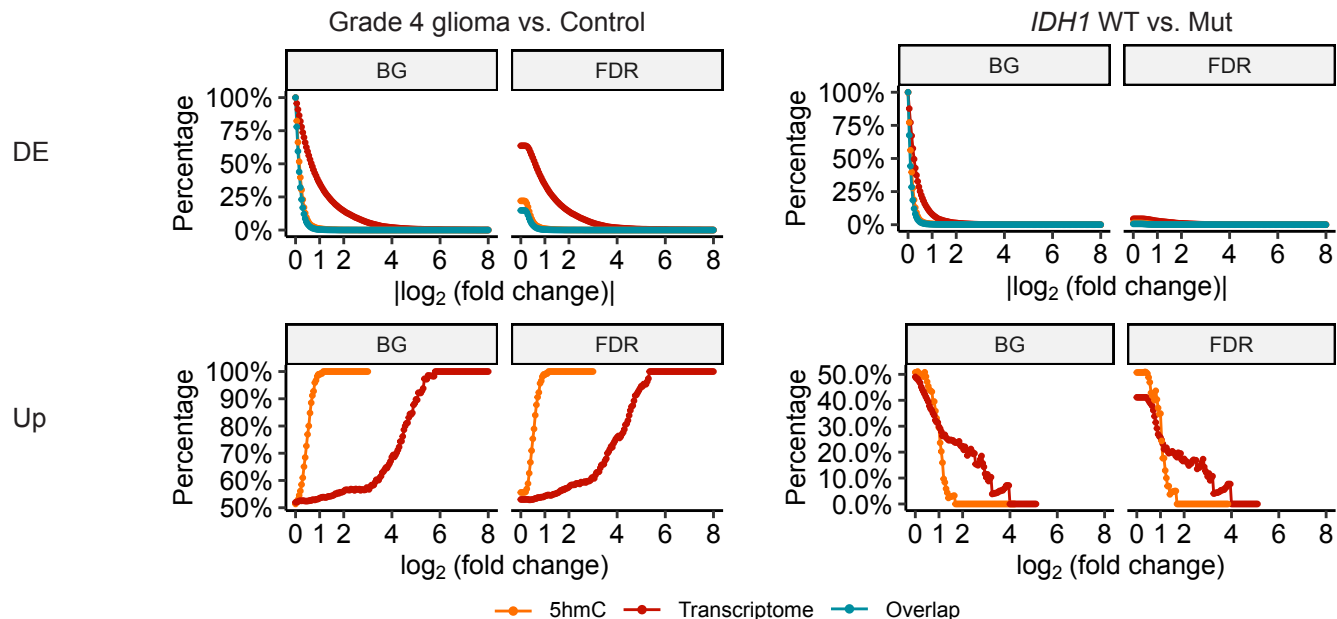

B

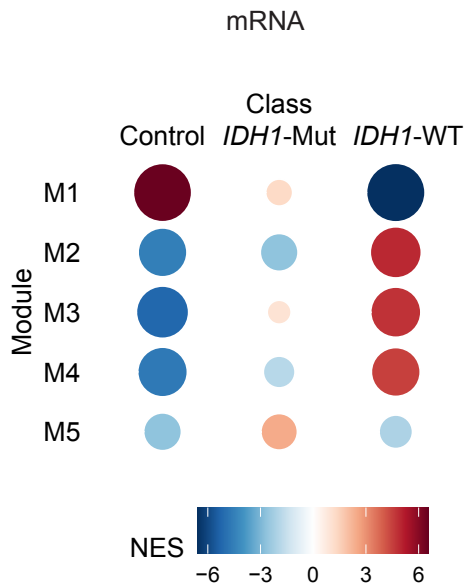

C

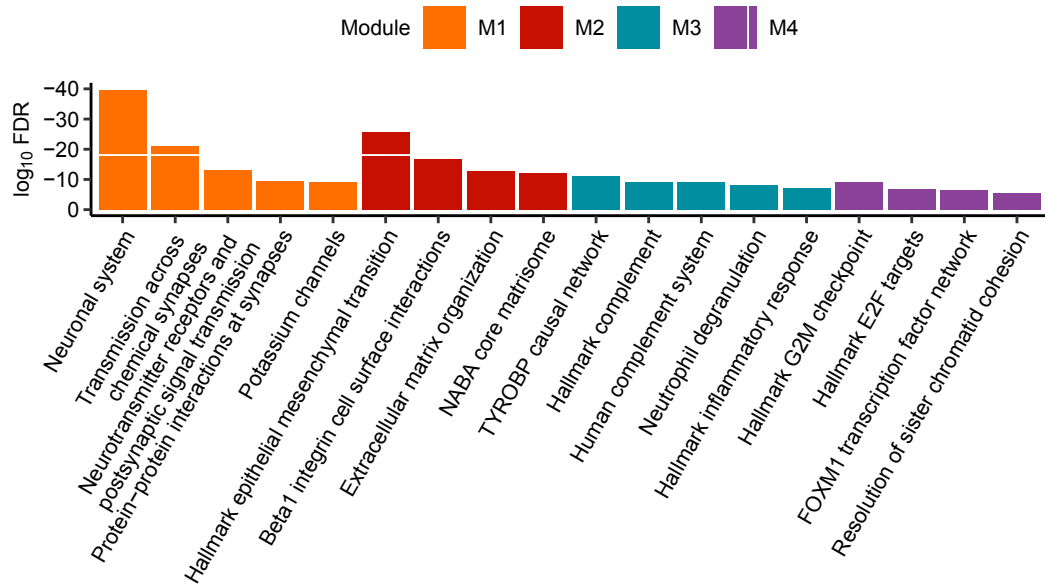

Supplement: Supplementary file 1 — Additional file 1. Fig. S1. (A) The percentages of genes with differential 5hmC modification and expression under different fold change cutoffs. BG denotes background gene; FDR denotes mRNA/lncRNA with FDR <0.05; Up: up-modified/expressed; down: down-modified/expressed. (B) Enriched co-regulated mRNA-mRNA (gene expression) modules are detected in normal controls, IDH1-Mut, and IDH1-WT tumors. (C) Top five enriched KEGG pathways associated with mRNA-mRNA co-regulated modules (FDR < 0.05 and gene count > 5) are shown. [file 40478_2023_1619_MOESM1_ESM.pdf]

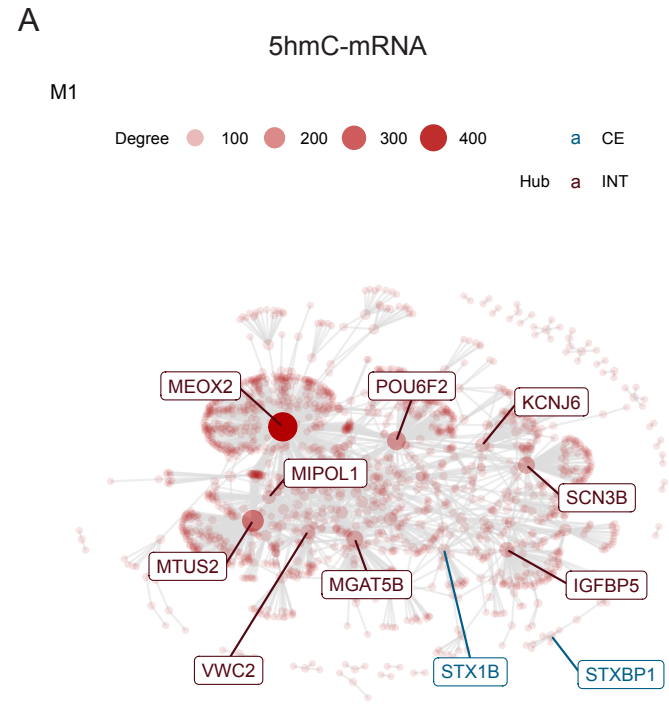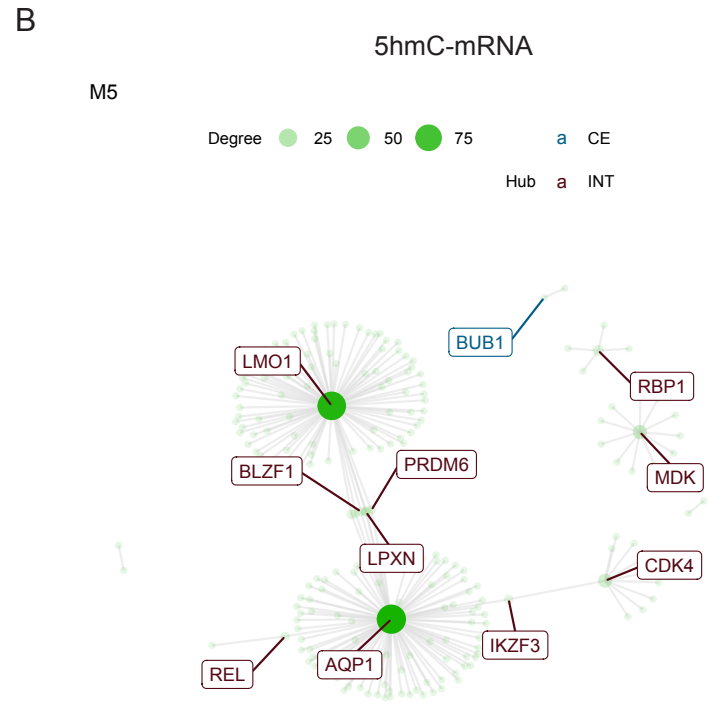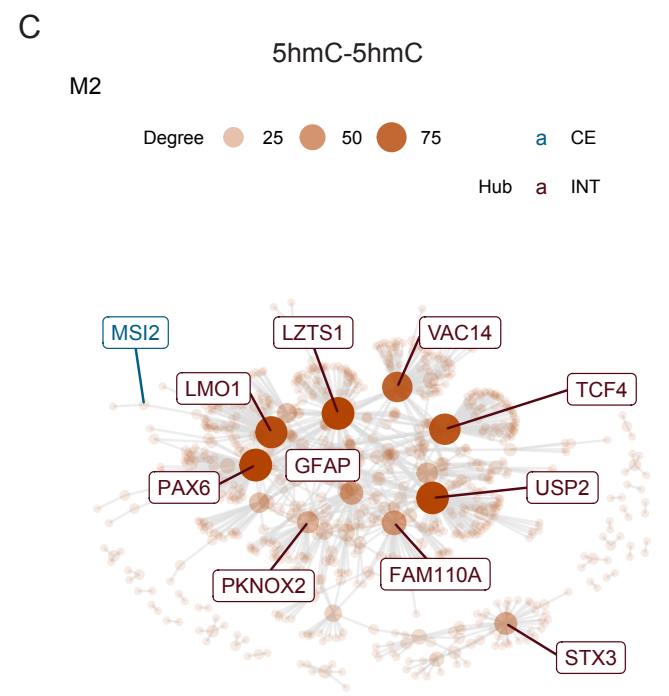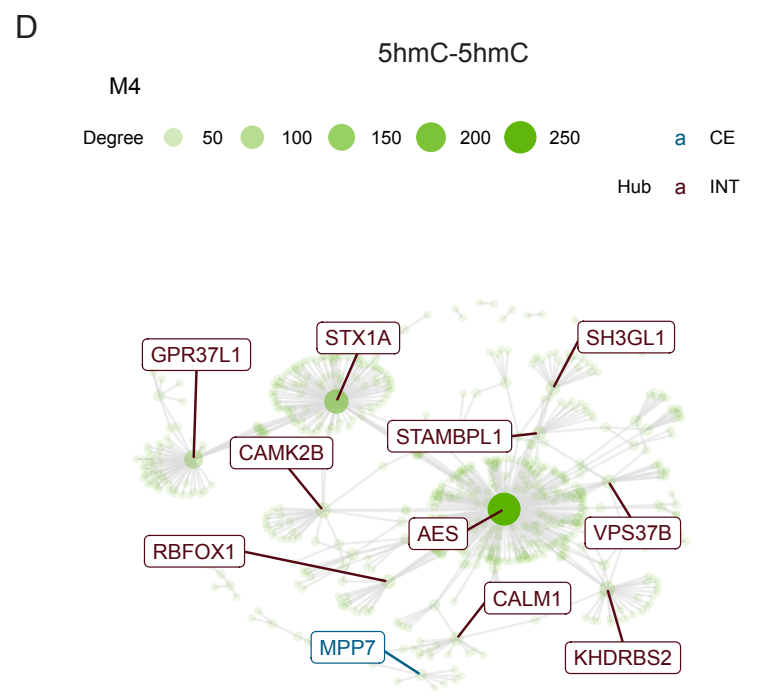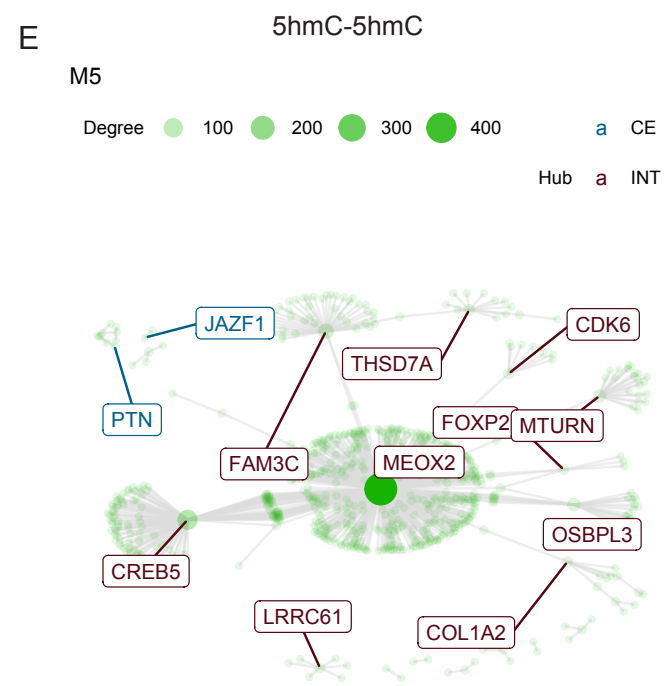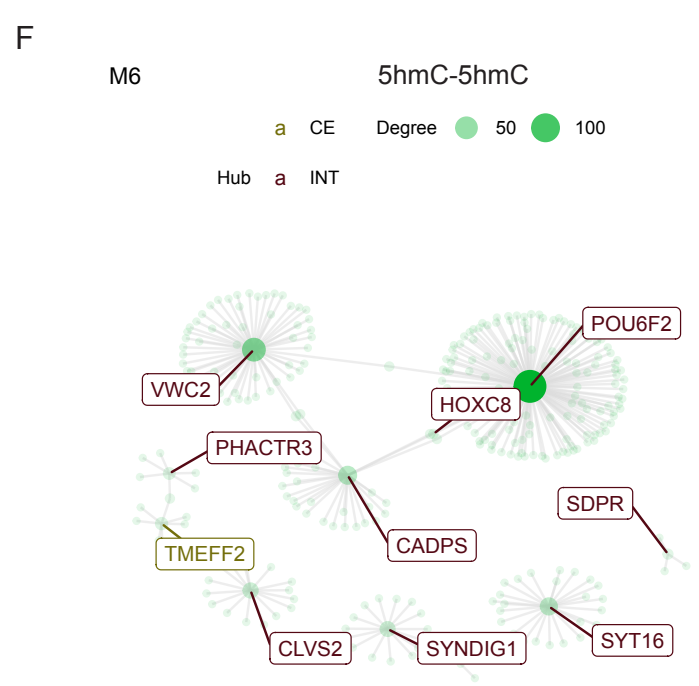

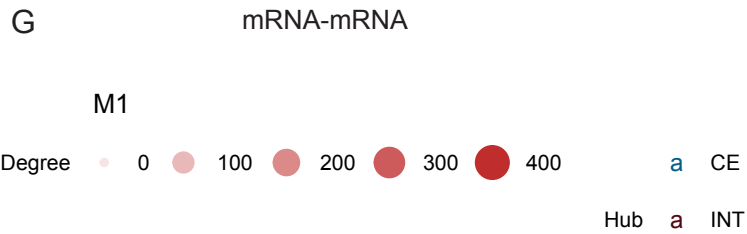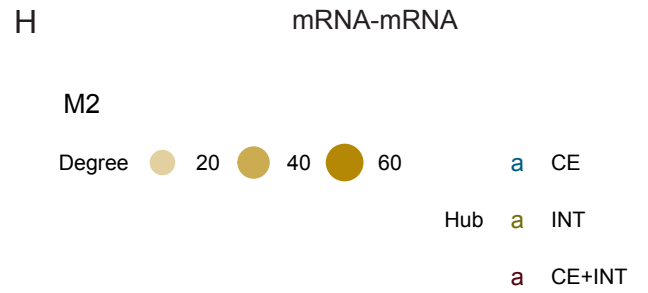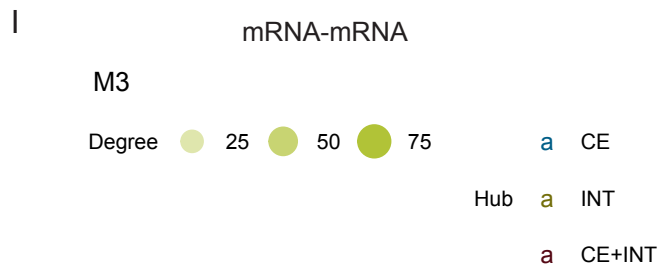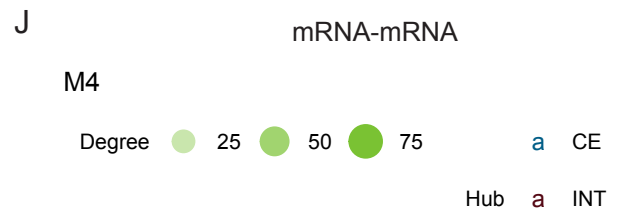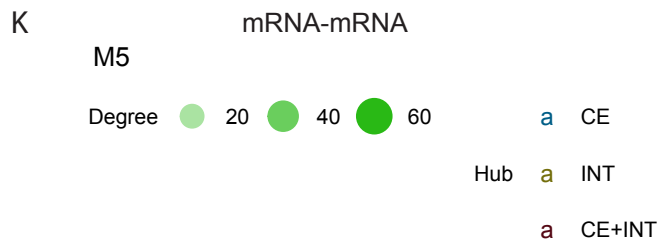

Supplement: Supplementary file 2 — Additional file 2. Fig. S2. (A) Network hubs of co-regulated 5hmC-mRNA module M1. (B) Network hubs of co-regulated 5hmC-mRNA module M5. (C-F) Network hubs of co-regulated 5hmC-5hmC module. (G-K) Network hubs of co-regulated mRNA modules. CE denotes co-expression/regulation hubs; INT denotes protein-protein interaction hubs; CE+INT denotes co-expression/regulation and protein-protein interaction hubs. [file 40478_2023_1619_MOESM2_ESM.pdf]

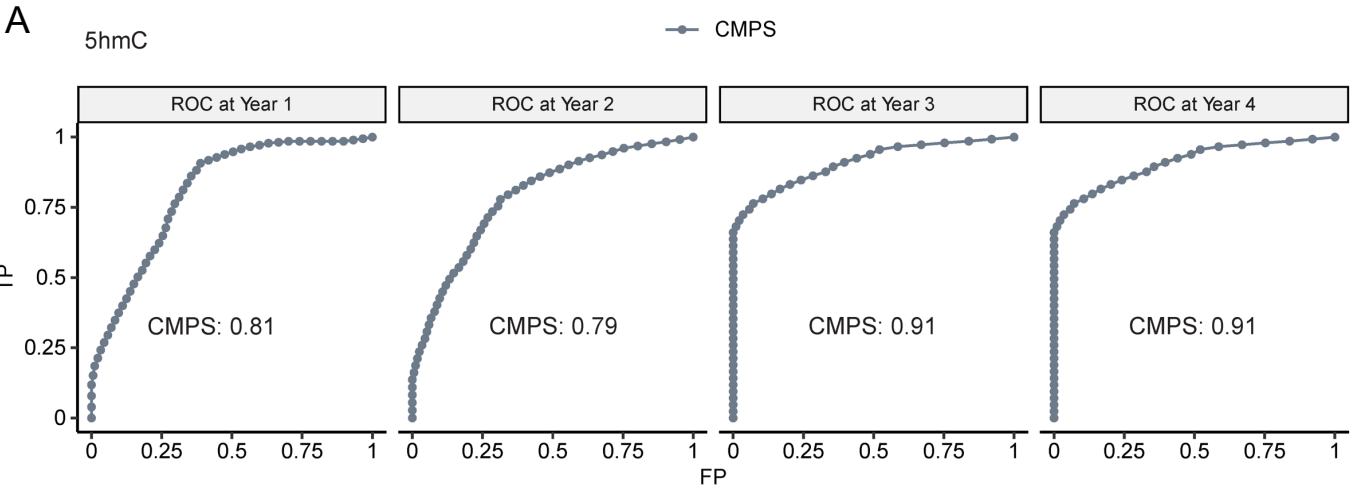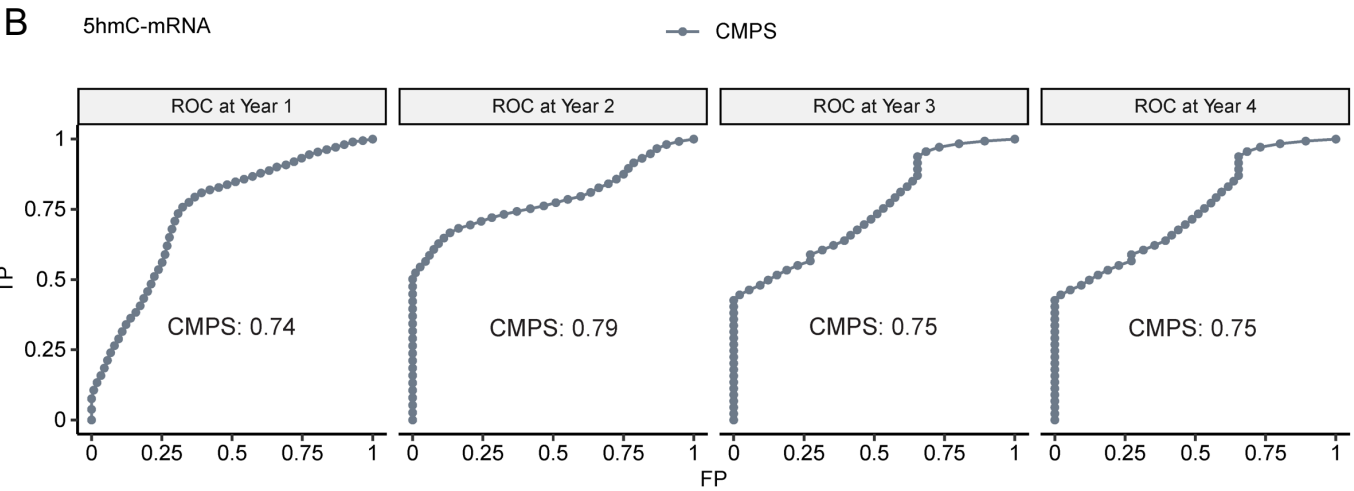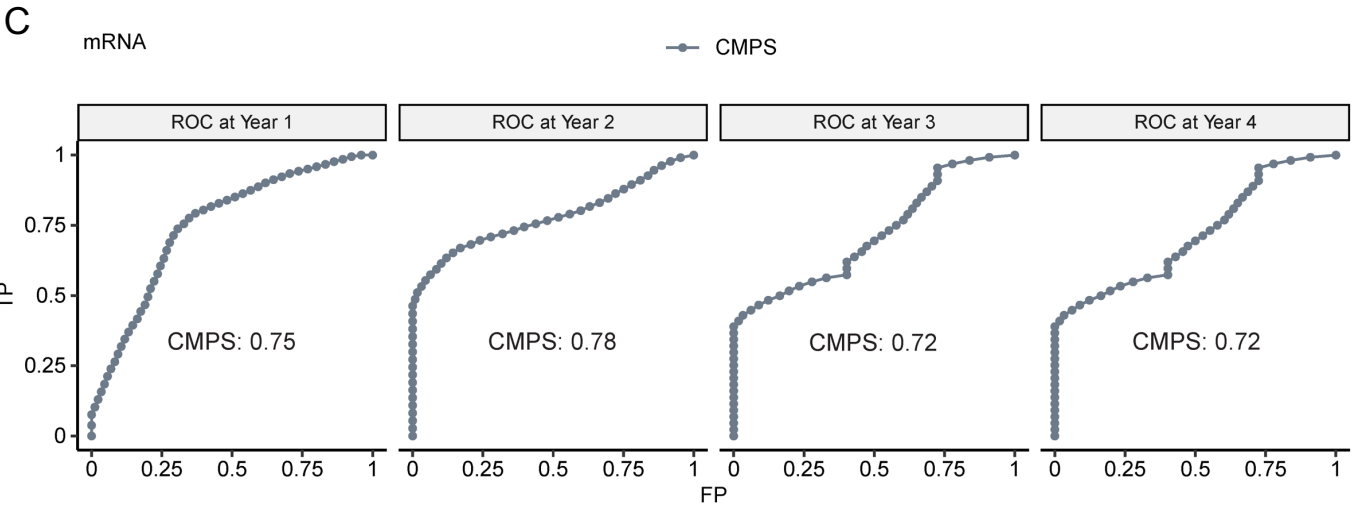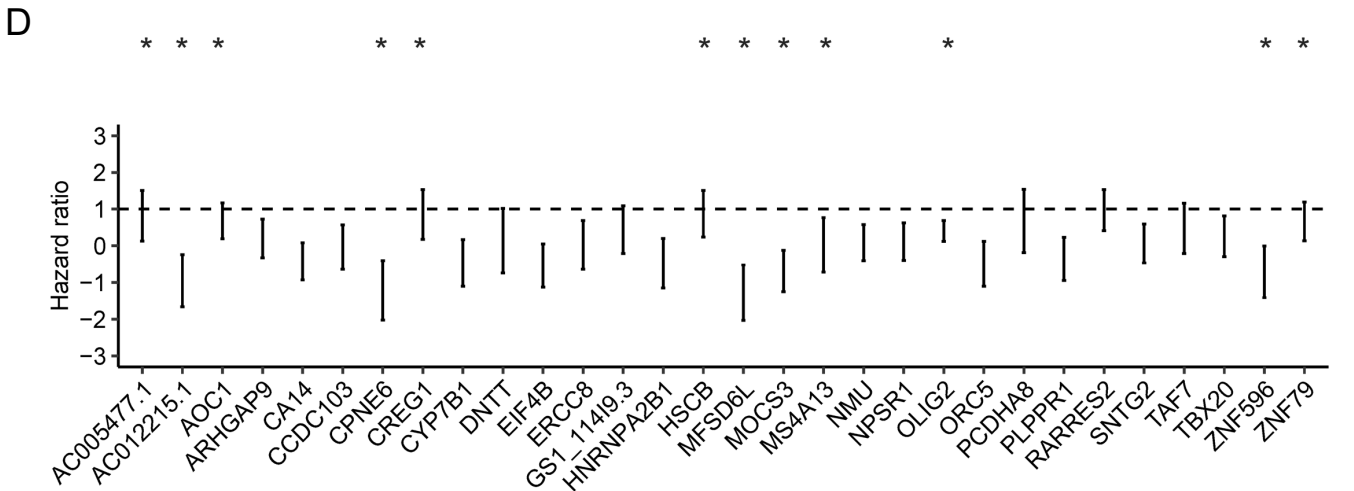

Supplement: Supplementary file 3 — Additional file 3. Fig. S3. Time-dependent ROC curves for IDH1-WT tumor patients’ survival with AUC measures evaluated using (A) 5hmC-5hmC co-regulated modules derived CMPS; (B) 5hmC-mRNA co-regulated modules derived CMPS; (C) mRNA-mRNA co-regulated modules derived CMPS. (D) Forest plots showing hazard ratios (HR) of the 30 promoters in the best-performed model. HR > 1 indicates increased survival risk per unit change in the 5hmC value; HR < 1 indicates decreased survival risk per unit change in the 5hmC value. [file 40478_2023_1619_MOESM3_ESM.pdf]
